# Initial resuscitation of burn patients: association between hemodynamic parameters and serum lactate level with 90-days mortality

**DOI:** 10.1186/2197-425X-3-S1-A849

**Published:** 2015-10-01

**Authors:** S Soussi, B Deniau, C Levé, A Ferry, V Maurel, M Benyamina, A Blet, B Le Cam, M Chaussard, I Iordache, M Mimoun, M Chaouat, A Mebazaa, M Legrand

**Affiliations:** Department of Anesthesiology and Critical Care, Saint-Louis Hospital, Burn Center, Paris, France; Department of Plastic Surgery, Saint-Louis Hospital, Burn Center, Paris, France

## Introduction

Initial resuscitation, historically based on crystalloids fluids administration, is essential to burn patients' survival. However, hemodynamic targets have been poorly explored although avoidance of both hypovolemic status and too liberal fluid administration is the bottom line [1, 2].

## Objectives

To evaluate the association between the first 24 hours after admission systemic hemodynamics, central venous saturation (ScvO_2_), central venous to arterial carbon dioxide difference (PCO_2_gap) and serum lactate with the 90-days mortality in critically ill burn patients.

## Methods

Burn patients with total body surface area (TBSA) greater than 20% admitted within 8 hours of thermal injury, with continuous cardiac output, ScvO_2_,PCO_2_gap and serum lactate monitoring during the first 24 hours after admission between march 2013 and October 2014, were included. Ringer Lactate was administered according to the Parkland formula (4ml/kg/% TBSA), and adjusted according to a local algorithm based on hemodynamic targets. Cardiac output was measured with the transpulmonary thermodilution method. All patients had invasive blood pressure monitoring. The primary study endpoint was 90-daysmortality. Results are indicated in median and (25-75) centiles. ANOVA test analysis or Mann-Whitney.

## Results

**Figure 1:**
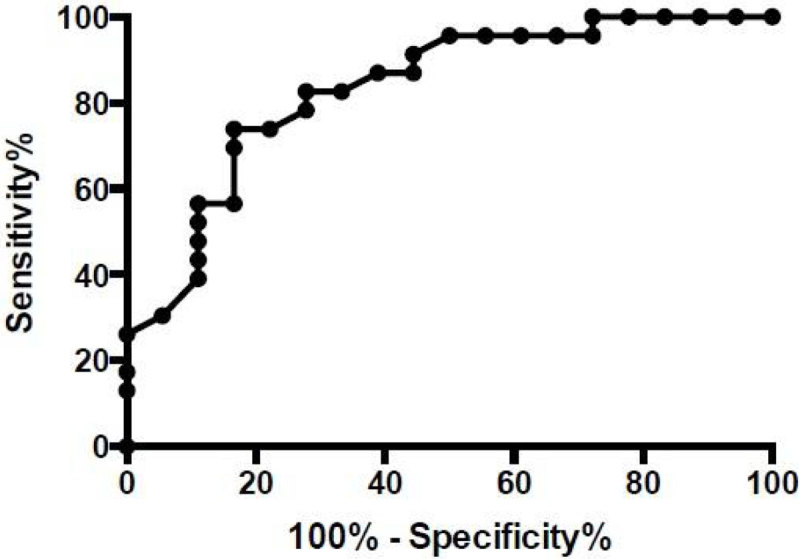
**Lactate ROC curve for 90days-mortality prediction**.

42 patients (27 men) were included, aged of 48 (34-58) years, with TBSA of 41 (29-56)%. 21%suffered from chronic arterial hypertension. SAPS II and ABSI were 30 (21-50) and 9(7-12) respectively. 40% presented with smoke inhalation injury. HbCO admission was 2(1-5)%. 92% were intubated and mechanically ventilated. 28 and 90 days mortality were 26 and 42% respectively. 40% presented acute kidney injury and 21% required renal replacement therapy in the first 7 days. Initial in-hospital Mean arterial pressure (MAP) and cardiac index (CI) were significantly lower in patient who died (68 vs 85 mmHg, p = 0.03 and 2.1 vs 2.7 L/min/m^2^). Thereafter, these differences became non significant. Increase in SOFA score between admission and 48 hours was associated with poor prognosis. No difference between initial urine output, central venous pressure, ScvO_2_ or PCO_2_gap was observed (0.4 vs 0.8 ml/Kg/h, 10 vs 9 mmHg, 80 vs 81% and 7 vs 8 mmHg respectively). Initial Serum lactate was significantly higher in patients who died (4.4 vs 2.2 mmol/L, p < 0,001). Area under the ROC curve for lactate was 0.83 (CI 0.7-0.95) to predict 90 days mortality (Figure 1).

## Conclusions

Admission serum lactate level in burn patients is a good biomarker to predict 90-days mortality. Initial CI and MAP seem to be lower in patients with poor prognosis but these differences disappeared with hemodynamic resuscitation. Our study suggests the necessary specific attention in pre-hospital phase and very early initial in-hospital resuscitation in severe burn patients.
